# Gaps in disability inclusion across universities in the United States

**DOI:** 10.1371/journal.pone.0317920

**Published:** 2025-01-22

**Authors:** Caroline Cerilli, Jiafeng Zhu, Varshini Varadaraj, Jessica Campanile, Fiona Sweeney, Jared Smith, Gayane Yenokyan, Bonnielin Swenor

**Affiliations:** 1 Disability Health Research Center, Johns Hopkins University School of Nursing, Baltimore, Maryland, United States of America; 2 Center for Education in Health Sciences, Northwestern University Feinberg School of Medicine, Chicago, Illinois, United States of America; 3 WebAIM, Institute for Disability Research, Policy and Practice, Utah State University, Logan, Utah, United States of America; 4 Biostatistics Center, Johns Hopkins University School of Public Health, Baltimore, Maryland, United States of America; University of Hyderabad, INDIA

## Abstract

Disabled people face social and environmental barriers to higher education, yet there is a dearth of clear, publicly available information on university websites related to accessibility and disability inclusion. Our team previously developed disability inclusion scores for the top 50 universities offering undergraduate programs based on funding from the National Institutes of Health (NIH) and found low scores overall. Building on that, this study examines the relationship between disability inclusion (as scores ranging from 0 to 100 points) and six university characteristics for these 50 universities. The six university characteristics examined were U.S. News National Universities 2021 rankings, geographic region, year of institution founding, total number of undergraduates, public or private status of the university, and presence of an undergraduate disability studies program. Results indicate that university characteristics were not significantly associated with disability inclusion scores (p>0.05 for all university characteristics). The average scores across all categories of each characteristic fell below 72 points. These results indicate a gap in disability inclusion across all types of universities examined. Highly funded universities should evaluate their campuses for disability inclusion.

## Introduction

Students determine college application choices and acceptance decisions based on a variety of university factors, including demographics of university enrollment, prestige, and proximity to home [1]. Universities have increasingly curated their online presence to market themselves to prospective students and use their admissions websites to highlight university values, history, and culture [2–4]. To express the value of diversity, many universities share information related to current or admitted student demographics, typically related to gender, race, sociodemographic information, and home country. Yet, data on disability inclusion are rarely represented in campus demographic breakdowns, and the absence of this information may signal that universities do not value disability as diversity [2,5,6]. Just 34% of disabled students who enroll in 4-year undergraduate programs graduate within 8 years of enrolling—nearly half the 6-year graduation rate of all first- and full-time students at the same programs (63%) [7,8]. In general, universities are not yet reaching out to disabled students.

University websites are a key form of communication for institutions to inform current and prospective students, faculty, and staff about accessibility and admissions processes [1]. While prospective students report that they prefer visiting universities to gain an understanding of the institution, this is often an expensive and time-consuming process [9]. Disabled students may have reduced ability to plan trips, as university websites are often unclear about campus and tour accessibility [6]. This, in conjunction with limitations on travel due to the COVID-19 pandemic, has led to an increase in reliance of prospective students on publicly available information provided by the institution to gain a sense of existing policies and university values. Transparent information about university accessibility is crucial to campus navigation.

More broadly, clear information about which institutions meet baseline criteria for accessibility is necessary to advance disability inclusion in education. To begin to bridge this gap, we developed a novel scorecard to measure accessibility and disability inclusion across the websites of the top 50 National Institutes of Health (NIH) funded undergraduate programs; results are reported publicly in the Disability Health Research Center (DHRC) Undergraduate Disability Inclusion Dashboard [6,10]. Through this dashboard, we found that most universities do a poor job of sharing information related to accessibility for disabled students. In this updated work, we sought to further understand if any university characteristics are associated with disability inclusion scores to help identify the types of universities that are leaders in disability inclusion, and those that require improvement.

## Methods

This study compared 50 universities’ scores from the Johns Hopkins Disability Health Research Center (JHU DHRC) University Disability Inclusion Dashboard (referred to as disability inclusion scores) with selected universities’ characteristics (2021). The methods to create the University Disability Inclusion Dashboard draw on similar dashboards, such as the Supplemental Nutrition Assistance Program accessibility (SNAP-Access) score dashboard and the Covid-19 Vaccine dashboard, which both assess and rank public services for accessibility [11,12]. Further, similar methods have been used to examine which large research university characteristics (such as university enrollment, prestige, public or private status, and geographic location) may be related to the establishment of campus Lesbian, Gay, Bisexual, Trans, and Queer (LGBTQ) centers, which serve another underserved group on campus [13]. This study relied on publicly available information and did not involve any human subjects, and therefore did not require Institutional Review Board approval.

### University selection

This study examined the top 50 NIH-funded undergraduate degree granting universities for the fiscal year of 2020. Funding data was collected from NIH RePORTER on June 21^st^, 2021, an online database which holds information regarding NIH funding awarded to institutions [14]. This selection criterion was used because the Rehabilitation Act of 1973 requires institutions receiving federal funding, such as from the NIH, to address and prevent discrimination on the basis of disability [15].

### University disability inclusion scores

This study used data from the JHU Disability Inclusion Dashboard, which examined universities for inclusive practices for disabled students [10] as previously described by Campanile et al. [6]. This dashboard is openly available at https://disabilityhealth.jhu.edu/inclusiondashboard/. In brief, data for ten indicators were abstracted from publicly available university websites from April 2021 to October 2021 under four categories of disability inclusion: accessibility, public image, accommodations process, and grievance policy, totaling 100 points. Indicators assessed the availability of information regarding: built environment, virtual environment, disability accommodation office statements, statistics on disabled students, staff, and faculty, disability accommodation office contact information availability, confidentiality in the disability accommodation process, rights and responsibilities of students and disability service staff, rights and responsibilities of faculty, timeline regarding accommodations, and disability related grievance policies. The scores were used to determine overall university letter grades for inclusion as follows: 100–90 points received an A, 89–80 a B, 79–70 a C, 69–60 points a D, and 59 points or below an F.

As shown on the DHRC Dashboard, 3 universities received an A disability inclusion grade (100–90 points), 7 received a B (89–80 points), 10 received a C (79–70 points), 16 received a D (69–60 points), and 14 received an F (below 70 points). Additionally, the average 2020 NIH funding for the 50 universities was $284,936,928 [10].

### Examined university characteristics

As detailed in Campanile et al.[6], trained researchers collected data on six university characteristics of each of the 50 institutions from November 2021 to February 2022 to compare against the disability inclusion scores. All data were collected from the universities’ websites, NIH RePORTER, and the U.S. News webpages (S1 Appendix). The university characteristics examined were: (1) U.S. News National Universities 2021 ranking (categorized 1–10, 11–50, 51–100, 101–250), (2) geographic region of the institution (West, South, Northeast, Midwest), (3) total undergraduate enrollment in 2020 (small, medium, large), (4) year of founding (1800, 1800–1899, 1900), (5) if the university is public or private, and (6) the presence of an undergraduate Disability Studies program (yes or no). These characteristics were examined given their relevance to prospective students’ decisions around university selection and the data availability across all institutions. Additionally, these characteristics are influential of campus culture as perceived by students [1].

U.S. News is a major source of information which ranks universities based on alumni employment and debt, faculty experience, and institutional funding and resources [16]. These rankings have been shown to be a tool for prospective students in making informed decisions for selecting undergraduate programs, and they influence the prestige of institutions [17]. The U.S. News National Universities 2021 rankings list was used. This list is updated annually in September. We organized institutional rankings from U.S. News into four categories: 1 to 10, 11 to 50, 51 to 100, and 101 to 250. One university (Oregon Health Sciences University) was not represented on the U.S. News National Rankings 2021 list and was excluded from this category of analysis.

The geographic regions of the universities were designated as West, South, Northeast, and Midwest, defined by census map divisions [18]. Locations for universities were found on NIH RePORTER [14]. We wanted to evaluate if regional differences affected disability inclusion, as this may be attributed to socioeconomic status of geographic location, local legislation, or physical landscape distinctions.

Data on total undergraduate enrollment for Fall 2020 was gathered via the U.S. News webpages for each university [16]. One university (Oregon Health and Science University) did not list information on U.S. News regarding enrollment, and so data from university reports were used. In two cases (University of Washington and University of Pennsylvania), the university website reports for undergraduate enrollment differed from the U.S. News report, and data from U.S. News was used as it reflected updated estimates. We examined undergraduate population in three categories: small (≤5,000 undergraduates enrolled), medium (>5,000 and <15,000 undergraduates enrolled), and large (>15,000 undergraduates enrolled), informed by the Carnegie Classification of Institutions of Higher Education (n.d.).

The year of founding for each institution was also collected from their U.S. News webpages. Institutions were examined by centuries in three categories: 1800, 1800 to 1899, and 1900 to present. This characteristic was studied since the age of an institution and its physical landscape may be relevant to present day accessibility due to the development of modern architecture and the regulations of the Americans with Disabilities Act.

Data on whether universities were private or public was collected from U.S. News webpages for each institution. While each institution evaluated in this study receives federal funding from the NIH, there are differences in state and private funding across these two types of universities [19].

Data on presence of universities’ undergraduate disability studies programs (inclusive of major, minor, or certificate) was abstracted from university websites, such as admissions pages. Our definition of disability studies programs includes those that conceptualize disability through an interdisciplinary social, cultural, and rights lens; it excludes programs related to educating disabled students, such as in the case of special education, or medicalizing disabled people [20].

### Statistical analysis

Descriptive statistics including frequencies and percentages were used to summarize university characteristics. Kruskal-Wallis Rank Sum tests were used to compare the mean and standard deviation disability inclusion score by category for each university characteristic. Significance was set at H(1)>3.841, H(2)>5.991, H(3)>7.815, and p<0.05. Statistical analyses were completed using R statistical software (version 4.1.1).

## Results

### University characteristics

This study examined 50 universities in total, and the descriptive university characteristics are found in Table 1. Nine (18%) universities ranked 1 to 10 on the U.S. News National University 2021 report, 22 (44%) universities ranked 11 to 50, 7 (14%) universities ranked 51 to 100, and 11 (22%) universities ranked 101 to 250. This study included 13 (26%) universities in the Midwest, 11 (22%) universities in the Northeast, 14 (28%) universities in the South, and 12 (24%) universities in the West. Five (10%) institutions were small (≤5,000 undergraduates enrolled in 2020), 18 (36%) were medium-sized (5,000–15,000 undergraduates), and 27 (54%) were large (>15,000 undergraduates). Also, 7 (14%) institutions were founded prior to 1800, 34 (68%) institutions were founded 1800–1899, and 9 (18%) institutions were founded 1900 or later. The majority (N = 29, 58%) were public institutions and 12 (24%) institutions offered undergraduates a major, minor, or certificate in a disability studies program.

**Table 1:** Summary of total disability inclusion and accessibility scores by university characteristics for top 50 NIH funded undergraduate universities.

University Characteristic^a^	Category	N (%)	Mean (SD)^b^	H-value	P-value^b^
U.S. News National Universities Ranking, 2021					
	1 to 10	9 (18)	67 (11)	1.697	0.638
	11 to 50	22 (44)	64 (16)		
	51 to 100	7 (14)	69 (12)		
	101 to 250	11 (22)	60 (14)		
Geographic Region					
	Midwest	13 (26)	61 (13)	1.517	0.678
	Northeast	11 (22)	63 (16)		
	South	14 (28)	64 (15)		
	West	12 (24)	68 (14)		
Founding Year					
	<1800	7 (14)	64 (9)	0.563	0.755
	1800–1899	34 (68)	64 (15)		
	≥1900s	9 (18)	63 (14)		
Undergraduate Population					
	Small (<5,000)	5 (10)	54 (18)	2.475	0.290
	Medium (5,000–15,000)	18 (36)	64 (15)		
	Large (>15,000)	27 (54)	65 (13)		
Public or Private					
	Private	21 (42)	63 (16)	0.001	0.976
	Public	29 (58)	65 (13)		
Disability Studies program					
	Yes	12 (24)	71 (12)	3.513	0.061
	No	38 (76)	62 (14)		

^a^Universities were organized into one category per characteristic and average scores were found within each category.

^b^Among the top 50 NIH funded institutions, no university characteristic shows a significant relationship to disability inclusion score. Kruskal-Wallis Rank Sum tests were used to compare the mean and standard deviation disability inclusion score by category for each university characteristic, and p>.05 for all characteristics.

### Association between university characteristics and disability inclusion

**Fig 1:**
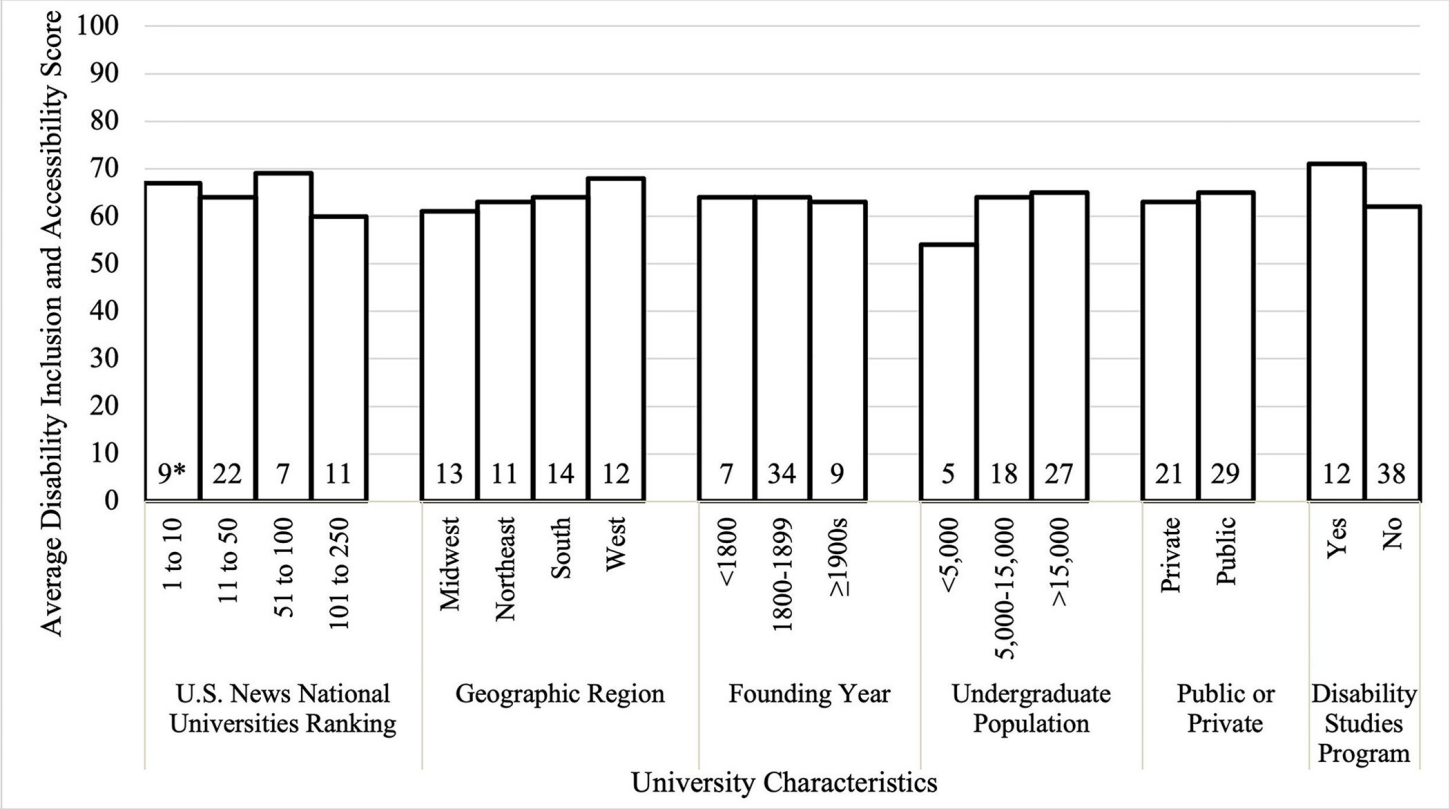
Average university disability inclusion and accessibility scores within each studied university characteristic for the top 50 NIH funded universities of 2020. ^a^Values at bottom of bars represent number of universities studied in each subcategory. Average disability inclusion scores are not statistically significantly different within university characteristics. We sorted universities into categories across six characteristics and found the average disability inclusion scores (Campanile et al., 2022) within each category. Kruskal-Wallis Rank Sum tests were used to compare the mean and standard deviation disability inclusion score by category for each university characteristic, and p>.05 for all characteristics.

There were no statistically significant associations found between the university characteristics examined and the disability inclusion scores of the universities, (p>0.05 for all), (Table 1, Fig 1).

Universities ranked 1 to 10 on the U.S. News National University rankings 2021 report averaged 67 points (SD = 11), those ranked 11 to 50 averaged 64 points (SD = 16), those ranked 51 to 100 averaged 69 points (SD = 12), and those ranked 101 to 250 averaged 60 points (SD = 14). Midwestern universities averaged 61 points (SD = 13), Northeastern universities averaged 63 points (SD = 16), Southern universities averaged 64 points (SD = 15), and Western universities averaged 68 points (SD = 14). Regarding university population size, small (<5,000 undergraduates), medium (5,000–15,000 undergraduates), and large (>15,000 undergraduates) institutions averaged 54 (SD = 18), 64 (SD = 15), and 65 points (SD = 13) respectively. Institutions founded prior to 1800 averaged 64 points (SD = 9), institutions founded 1800 to 1899 averaged 64 points (SD = 15), and institutions founded in 1900 to present averaged 63 points (SD = 14). Public universities averaged 65 points (SD = 13), and private universities averaged 63 points (SD = 16). Institutions that offer disability studies averaged 71 points (SD = 12), whereas institutions without any such disability studies program averaged 62 points (SD = 14).

## Discussion

Our study found that gaps in disability inclusion and accessibility are pervasive across the top funded universities in the US, regardless of university characteristics including national rankings, region, size, year of founding, and public vs private status of the university. These findings demonstrate the need for leadership at universities of all types to direct greater attention and resources towards disability inclusion and accessibility.

There is limited quantitative research available on disability inclusion across higher education; however, existing work indicates that disabled students consistently face lower access to education due to systemic ableism [21–23]. A policy analysis across 193 countries found that just 54% of countries have legislation against discrimination on the basis of disability in primary through secondary education, 75% provide disabled students access to desegregated education through secondary education, and 65% guarantee disabled students academic accommodations through secondary education—despite the wide adoption of the United Nations’ recognitions of disabled students’ rights to education [24]. Without access to foundational education, disabled students are set at a disadvantage. In the U.S., as of 2019, approximately half of public and private non-profit universities represented less than 3% disabled students in their student populations [25]. Qualitative work, while often limited to one or few universities at a time, has also revealed disabled undergraduate students’ barriers to education and social opportunities [26,27]. Disabled students are not provided tools to assess a university for disability inclusion, and many apply to and attend universities that do not meet their access needs; many are discouraged entirely from applying to higher education at all [28]. Major issues include student disability service offices that often do not provide enough support to students to obtain accommodations, nor do they guarantee high quality accommodations; further, faculty may impede a student’s ability to engage with a class through refusing to support accommodations [29]. Despite these documented inequities, there is little standardized data across undergraduate education related to disability.

In contrast, our study found a widespread lack of disability inclusion across U.S. universities of various kinds. This type of standardized data across universities is critical for measuring change as higher education considers what future inclusion looks like. Universities are increasingly collecting information on campus diversity, such as racial and gender demographics of the student body, yet there remains a scarcity of quantitative data on and attention to disability inclusion. Data on campus diversity is essential to pushing universities beyond mission statements and towards greater inclusion, especially when this data can be compared across universities [30,31]. With the help of racial demographic data collected from universities, U.S. News created the Campus Ethnic Diversity Dashboard to track and quantify hundreds of universities and colleges’ diversity index, where a higher score indicates greater racial diversity [32]. This project indicates current standards of racial diversity across the U.S. and shows where universities are most inclusive so students can make informed decisions when applying to and attending these institutions. Additionally, prior research has found that the establishment of LGBTQ centers on university campuses is associated with larger research universities, and in particular, greater university prestige, public university status, non-Southern located universities, and higher undergraduate enrollment [13]. This reveals that university characteristics are influential in other aspects of campus diversity and inclusion. Collection of data on disability for students, faculty, and staff makes comparison of universities possible [6,33]. Institutional disability data gathering and action continues to lag in times of increased attention to diversifying college campuses.

Presence of a Disability Studies program was the only characteristic we examined for association with disability inclusion score that approached statistical significance (p = 0.06). Disability Studies offers opportunities to highlight disability when working on culture change at universities [20]. While these programs have not been rigorously studied, it would make sense that their presence offer more opportunities for disabled students to find disabled mentors, and to allow greater room for funding to be directed towards examining disability. Work from professors of color has shown that mentorship from professors with shared identity is important to student success [34]. However, universities must be careful not to use Disability Studies programs to silo topics related to disability and disabled people, as this work is intersectional and disabled people should be found across departments. Further study is required to examine the role Disability Studies programs play in accessibility and inclusion on campuses.

This study highlights an urgent need for university leadership to consider disabled people in higher education and promote their success. No highly funded university is exempt from these efforts. Current standards for the higher education environment do not yet include systems of accountability to ensure that the basic rights of the disability community are met. Highly funded universities have a responsibility to set and meet standards of disability inclusion in part as a result of their global influence. The JHU DHRC University Disability Inclusion Dashboard offers universities one step towards improving their campus culture, policies, and practices. This work is a call to action to all university leadership to give greater consideration towards accessibility and disability inclusion on campus.

In conclusion, this study found that university characteristics are not associated with disability inclusion scores among the top 50 NIH funded undergraduate universities. U.S. News National University Rankings, geographic region, year of institution founding, total number of undergraduates, public or private status of the university, and presence of an undergraduate disability studies program do not account for differences in disability inclusion across campuses. These results suggest a need for universities to re-evaluate their role in creating disability inclusive campuses.

### Limitations

This study has limitations that should be considered when interpreting the results. Only university characteristics for the top 50 NIH funded undergraduate universities were examined, which does not include many liberal arts colleges and community colleges. Future research should examine how this compares to different types of higher education, such as liberal arts education and community colleges. One study from 2013 found many community colleges fail to adhere to federal web accessibility standards, suggesting an urgency for this data [35]. Furthermore, this work only examined publicly available information. We relied on the same level of access to information that people not associated with universities (such as prospective students and faculty or staff members) would have. For greater work to be done, universities must increase transparency in their policies on disability inclusion. Additionally, this study was limited to six publicly available university criteria, which could be expanded to include others in future work. It is also important to note that disability inclusion scores are based on publicly available information, which does not assure a safe or inclusive campus experience for disabled students. However, these scores are an indication of research universities’ public forethought for disabled people.

## Supporting information

S1 Appendix University disability inclusion grades and scores, location, 2021 U.S. News rankings, and characteristics. (DOCX)
