# Supplementary material for: Gaps in disability inclusion across universities in the United States
Source: PLoS One. 2025 Jan 22;20(1):e0317920. doi: 10.1371/journal.pone.0317920 (PMC11753627; doi:10.1371/journal.pone.0317920)
Supplement: S1 Appendix — (DOCX) [file pone.0317920.s001.docx]

**S1 Appendix**

University disability inclusion grades and scores, location, 2021 U.S. News rankings, and characteristics

| **University^a^** | **Overall Disability Inclusion Grade^b^** | **Total Score^b^** | **Location^a^** | **U.S. News National Universities 2021 Ranking^c^** | **Undergraduate enrollment^c^** | **Institution founding year^c^** | **Public or private status^c^** | **Disability Studies program presence^d^** |
| --- | --- | --- | --- | --- | --- | --- | --- | --- |
| Johns Hopkins University | D | 65 | Baltimore, MD | 9 | 6,331 | 1876 | Private | no |
| University Of California Los Angeles | A | 90 | Los Angeles, CA | 20 | 31,636 | 1919 | Public | yes |
| University Of Michigan At Ann Arbor | F | 50 | Ann Arbor, MI | 23 | 31,329 | 1817 | Public | no |
| Duke University | D | 60 | Durham, NC | 9 | 6,717 | 1838 | Private | no |
| University Of Pennsylvania | C | 70 | Philadelphia, PA | 8 | 9,872 | 1740 | Private | no |
| University Of Pittsburgh At Pittsburgh | D | 60 | Pittsburgh, PA | 59 | 19,197 | 1787 | Public | no |
| Stanford University | B | 85 | Stanford, CA | 6 | 6,366 | 1885 | Private | no |
| Yale University | F | 50 | New Haven, CT | 5 | 4,703 | 1701 | Private | no |
| Washington University | D | 60 | Saint Louis, MO | 14 | 7,653 | 1853 | Private | no |
| University Of Washington | B | 85 | Seattle, WA | 59 | 35,582 | 1861 | Public | yes |
| University Of California, San Diego | F | 55 | La Jolla, CA | 34 | 31,842 | 1960 | Public | no |
| Univ Of North Carolina Chapel Hill | D | 60 | Chapel Hill, NC | 28 | 19,399 | 1789 | Public | no |
| Emory University | C | 70 | Atlanta, GA | 21 | 7,010 | 1836 | Private | no |
| Northwestern University At Chicago | C | 70 | Chicago, IL | 9 | 8,194 | 1851 | Private | no |
| University Of Minnesota | D | 60 | Minneapolis, MN | 68 | 36,061 | 1851 | Public | no |
| University Of Wisconsin-Madison | D | 60 | Madison, WI | 42 | 33,585 | 1848 | Public | yes |
| University Of Alabama At Birmingham | B | 80 | Birmingham, AL | 148 | 13,878 | 1969 | Public | no |
| University Of Southern California | C | 70 | Los Angeles, CA | 27 | 19,606 | 1880 | Private | no |
| Oregon Health & Science University | F | 50 | Portland, OR | N/A | 809 | 1887 | Public | no |
| University Of Colorado Denver | F | 55 | Aurora, CO | 227 | 14,995 | 1912 | Public | no |
| University Of California At Davis | D | 60 | Davis, CA | 38 | 31,162 | 1905 | Public | no |
| University Of Chicago | B | 80 | Chicago, IL | 6 | 6,989 | 1890 | Private | no |
| Ohio State University | D | 60 | Columbus, OH | 49 | 46,984 | 1870 | Public | yes |
| Indiana Univ-Purdue Univ At Indianapolis | D | 60 | Indianapolis, IN | 196 | 20,966 | 1969 | Public | no |
| University Of Utah | C | 70 | Salt Lake City, UT | 99 | 24,643 | 1850 | Public | yes |
| Case Western Reserve University | F | 40 | Cleveland, OH | 42 | 5,433 | 1826 | Private | no |
| University Of Iowa | B | 85 | Iowa City, IA | 83 | 22,304 | 1847 | Public | yes |
| University Of Florida | F | 55 | Gainesville, FL | 28 | 34,931 | 1853 | Public | yes |
| University Of Rochester | D | 60 | Rochester, NY | 34 | 6,521 | 1850 | Private | yes |
| University Of Virginia | A | 90 | Charlottesville, VA | 25 | 17,311 | 1819 | Public | no |
| University Of California-Irvine | F | 50 | Irvine, CA | 36 | 29,638 | 1965 | Public | no |
| University Of Arizona | C | 70 | Tucson, AZ | 103 | 36,503 | 1885 | Public | no |
| University Of Kentucky | C | 70 | Lexington, KY | 127 | 22,227 | 1865 | Public | no |
| University Of Illinois At Chicago | D | 65 | Chicago, IL | 103 | 21,921 | 1965 | Public | yes |
| University Of California Berkeley | C | 70 | Berkeley, CA | 22 | 30,980 | 1868 | Public | yes |
| Brown University | D | 65 | Providence, RI | 14 | 6,792 | 1764 | Private | no |
| Vanderbilt University | F | 35 | Nashville, TN | 14 | 7,057 | 1873 | Private | no |
| Massachusetts Institute Of Technology | D | 60 | Cambridge, MA | 2 | 4,361 | 1861 | Private | no |
| Tulane University Of Louisiana | F | 45 | New Orleans, LA | 42 | 7,700 | 1834 | Private | no |
| University Of Texas, Austin | B | 85 | Austin, TX | 38 | 40,048 | 1883 | Public | yes |
| Cornell University | A | 90 | Ithaca, NY | 17 | 14,743 | 1865 | Private | no |
| Dartmouth College | B | 80 | Hanover, NH | 13 | 4,170 | 1769 | Private | no |
| Virginia Commonwealth University | D | 65 | Richmond, VA | 172 | 21,943 | 1838 | Public | yes |
| Michigan State University | F | 55 | East Lansing, MI | 83 | 38,491 | 1855 | Public | no |
| University Of Cincinnati | F | 45 | Cincinnati, OH | 148 | 28,657 | 1819 | Public | no |
| Harvard University | D | 60 | Cambridge, MA | 2 | 5,222 | 1636 | Private | no |
| George Washington University | C | 70 | Washington, DC | 63 | 11,762 | 1821 | Private | no |
| University Of South Florida | F | 50 | Tampa, FL | 103 | 38,579 | 1956 | Public | no |
| Thomas Jefferson University | F | 30 | Philadelphia, PA | 148 | 3,783 | 1824 | Private | no |
| Temple Univ Of The Commonwealth | C | 70 | Philadelphia, PA | 103 | 27,306 | 1884 | Public | no |

^a^University names and locations were found on the National Institutes of Health RePORTER in November 2021 and selected for receiving top NIH funding.

^b^Overall disability inclusion grades and total scores are from the Johns Hopkins University Disability Health Research Center University Disability Inclusion Dashboard, available at <https://disabilityhealth.jhu.edu/inclusiondashboard/>.

^c^U.S. News National Universities 2021 rankings, university enrollments, and public or private status data were drawn from U.S. News webages from November 2021 to February 2022.

^d^Presence of disability studies for undergraduates data were found on websites hosted by the university examined from November 2021 to February 2022.
